# Predicting qualitative phenotypes from microarray data – the Eadgene pig data set

**DOI:** 10.1186/1753-6561-3-S4-S13

**Published:** 2009-07-16

**Authors:** Christèle Robert-Granié, Kim-Anh Lê Cao, Magali SanCristobal

**Affiliations:** 1 INRA, UR631 Station d'Amélioration Génétique des Animaux, F-31326 Castanet-Tolosan, France; 2 INRA, UMR444 Laboratoire de Génétique Cellulaire, F-31326 Castanet-Tolosan, France

## Abstract

**Background:**

The aim of this work was to study the performances of 2 predictive statistical tools on a data set that was given to all participants of the Eadgene-SABRE Post Analyses Working Group, namely the Pig data set of Hazard et al. (2008). The data consisted of 3686 gene expressions measured on 24 animals partitioned in 2 genotypes and 2 treatments. The objective was to find biomarkers that characterized the genotypes and the treatments in the whole set of genes.

**Methods:**

We first considered the Random Forest approach that enables the selection of predictive variables. We then compared the classical Partial Least Squares regression (PLS) with a novel approach called sparse PLS, a variant of PLS that adapts lasso penalization and allows for the selection of a subset of variables.

**Results:**

All methods performed well on this data set. The sparse PLS outperformed the PLS in terms of prediction performance and improved the interpretability of the results.

**Conclusion:**

We recommend the use of machine learning methods such as Random Forest and multivariate methods such as sparse PLS for prediction purposes. Both approaches are well adapted to transcriptomic data where the number of features is much greater than the number of individuals.

## Background

Often, an important goal of transcriptomic analyses is to identify differentially expressed genes; the expression level of each gene is explained by the phenotype in a linear model setting (either regression or ANOVA for a quantitative or a qualitative phenotype). Another important goal is to find biomarkers, *i.e. *genes that have a high predictive value for the phenotype. One statistical method that can be considered is discriminant analysis, where the phenotype is modelled as a linear combination of a subset of gene expressions. However, in the case of transcriptomic data, gene expressions are highly correlated, leading to multicollinearity problems in discriminant analysis. Furthermore, the high number of variables limits the use of this method. To circumvent this problem, one can pre-select the variables, or use approaches that can deal with a large number of variables.

In this study, the objective was to assess the behaviour of 2 prediction methods on one data set [1,2], where the phenotype is a set of factors, genotype or breed (Large White denoted LW, and Meishan denoted MS), and treatment (control and ACTH, coded c and a). Expression levels were available for 3686 genes on 24 animals. We first focus on the machine learning approach Random Forest (RF), and then on the modelling approach sparse Partial Least Squares (sPLS) to analyse this data set.

## Methods

### Random forests

Random forests [2] is a classification algorithm that takes advantage of the unstable property of Classification and Regression Trees (CART) classifiers [3] and their lack of accuracy by aggregating them. It combines two sources of randomness that improve the prediction accuracy: bagging (bootstrap aggregating) and random feature selection to construct each CART. This results in a low correlation of the individual trees as well as low bias and low variance. The individual trees T_k _are constructed as follows:

- N bootstrap samples (B_1_,..., B_N_) are drawn from the original data.

- Each sample B_k _(k = 1,..., N) is used as a training set to construct an unpruned tree T_k_. Let *p *be the input variables of the tree. For each node of T_k_, *m *variables are randomly selected (*m*<<*p*) to determine the decision at the node, where *m *is constant during the forest growing. Then the best split among these *m *predictors is chosen to split the node.

The predictions of the N trees are then aggregated to predict new data by majority vote for classification or by average for regression. Random forest avoids the need to perform a separate cross validation test to estimate the prediction error of the forest when performing a classification or a regression. While the forest is constructed, it generates an internal estimation of the generalisation error as follows:

- While constructing each tree T_k_, about one-third of the cases are left out of the bootstrap sample and are not used in its construction. These data are called "Out-of-bag" or OOB data and are used as an "internal" test set for each tree that is grown.

- The OOB predictions are then aggregated and the error rate, called "OOB error estimate" is computed for the whole forest and should lead to an accurate and unbiased generalisation error [2].

The Mean Decrease Accuracy measure was used in this study as a feature selection criterion, where the OOB data are used to obtain estimates of variable importance by evaluating their contribution to the prediction accuracy. The values of each variable in the OOB cases are randomly permuted and are run along the tree. The proportion of cases in the correct classes with permuted OOB data is then subtracted from the proportion of cases in the correct classes where OOB data have not been permuted. The Mean Decrease Accuracy averages the difference between these two accuracies over all trees in the forest and normalizes it by the standard error.

### PLS and Sparse PLS

Let Y be the *n *× *q *matrix of phenotypes with the 4 columns indicating each combination (LWa, LWc, MSa, MSc) for the 24 animals, and let X be the *n *× *p *matrix of the 3686 gene expressions measured on the 24 animals. Partial Least Squares regression (PLS) was introduced by Wold [4], first with the NIPALS algorithm and then followed by numerous variants. The PLS is based on the simultaneous decomposition of the data sets X and Y into loading vectors and associated latent variables. The main idea is to perform successive regressions with projections onto latent structures to highlight hidden or latent underlying biological effects. As in Principal Component Analysis (PCA), the PLS components (latent variables) are linear combinations of the initial variables. However, the coefficients that define these components are not linear, as they are solved via successive local regressions on the latent variables. Furthermore, PLS goes beyond a simple regression problem, since X and Y are simultaneously modelled by successive decompositions. The objective function involves maximizing the covariance between each linear combination of the variables from both groups:



The loading vectors are the *p- *and *q- *dimensional vectors u_h _and v_h _for each PLS dimension *h *and are respectively associated to the X and Y data sets. The associated latent variables are defined as *ξ*_h _= Xu_h _and *ω*_h _= Yv_h_. As in PCA, the loading vectors u_h _and v_h _are directly interpretable, as they indicate the importance of the variables from both data sets in relation with each other. The latent variables *ξ*_h _and *ω*_*h*_, that are *n*-dimensional vectors contain the information regarding the similarities or dissimilarities between the individuals or samples [5]. PLS is an iterative method that is suitable for high dimensional data sets and has a valuable stability property. However, this interesting approach does not allow feature selection, which renders the results difficult to interpret in the *n<<p *problem. Lê Cao *et al. *[6] proposed a sparse version of the PLS, that combines variable selection and modelling in a one-step procedure for such problems. The sparse PLS (noted sPLS) is based on Lasso regression [7] that penalizes the loading vectors using Singular Value Decomposition to solve the PLS [8].

Two criteria were used to select the dimension size *H *and the number of predictive genes to select on each dimension of sPLS: the Root Mean Squared Error Prediction (RMSEP), and the Qh^2 ^that measures the marginal contribution of each latent variable to the predictive power of the PLS model. Briefly, 1-Qh^2 ^is the ratio of the average PRediction Error Sum of Squares to the average of the Residual Sum of Squares, over the variables (refer to [9] for more details).

The methods used to analyse this data set are implemented in the R   software (RandomForest R package [10] , the R package "integrOmics" for sPLS  is available at www.math.univ-toulouse.fr/biostat).    

## Results

### Random Forest

Random Forest (RF) does not require fine-tuning of its parameters. In this study, however, random forest classifications with 10000 trees were performed in order to obtain stable results. When applied to the whole data set (3686 genes), RF gave a reasonably high prediction power, with an OOB estimate of error rate equal to 12.5%. After a pre-selection of differentially expressed genes (662 genes with a FDR < 20%), the prediction was perfect (with 0% of the OOB estimate of error rate).

**Figure 1:**
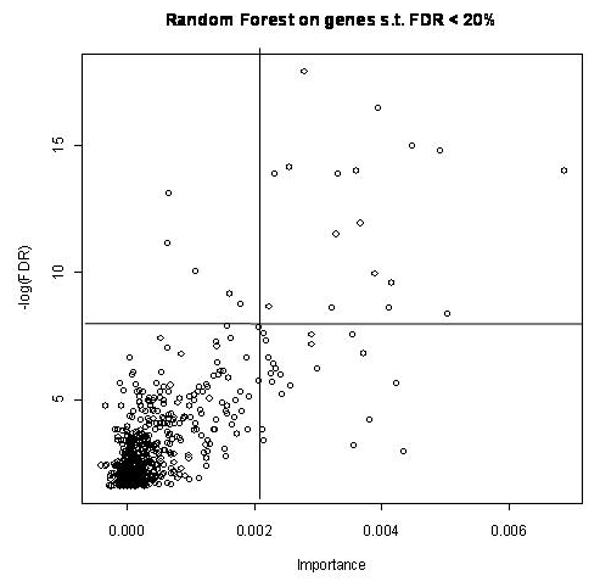
**Comparison of significance level (-log10 of the p-value in the differential analysis) with the importance measure of Random Forest**. The genes above the horizontal line are differentially expressed genes (t test) whereas the genes on the right hand side of the vertical line are declared as most important and highly predictive by Random forest.

Comparing the significant level of genes (-log10 of the p-value of the Fisher test in the differential analysis performed in [1]) with the importance given by RF (Mean Decrease Accuracy measure), we obtained a relatively high correlation between both measures (Figure 1). However, some of the most significant genes were not the most important (and vice-versa). Horizontal and vertical lines in Figure 1 were drawn to highlight the differences in the selections performed with the Fisher test or the Random Forest. Generally, this high correlation between the results of these two approaches was not encountered in other data sets [11], and may be explained here by the high proportion of differential genes with additive effects on genotype and treatment.

**Figure 2:**
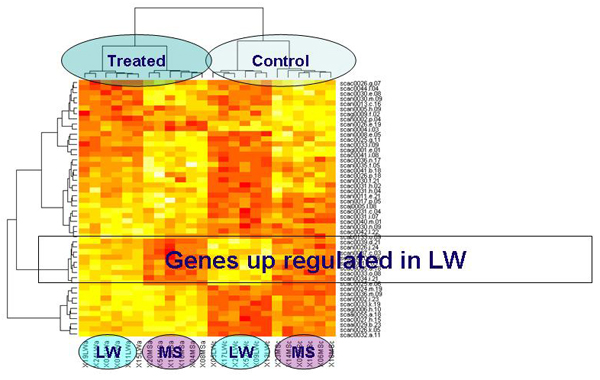
**Heat map displays of the hierarchical clustering results**. The light (dark) colour represents over-expressed (under-expressed) genes. The clusterings were performed with the Ward method and Euclidian distance with the 50 genes selected with Random Forest. Genes are displayed in lines and individuals in columns.

Hierarchical clustering is widely used as a statistical tool for microarray data to look for similarities between genes and samples in an unsupervised way. Hierarchical clustering using the Ward method and Euclidian distance [12] were thus used to evaluate the classification performances of the gene selection. The 50 most important genes were extracted to perform a heatmap (Figure 2). They allowed for a perfect classification of treated vs. control groups, and in each group the 2 genotypes were also clearly separated. Several clusters of genes appeared, e.g. a cluster of genes up regulated in treated animals (bottom), another in LW animals (2^nd ^cluster from bottom).

### PLS and sPLS

Recall that Y is the phenotype matrix of the 4 indicators (LWa, LWc, MSa, MSc) for each animal. The number of dimensions *H *to be retained was estimated with the Qh^2 ^criterion, for which a value below the threshold 0.0975 indicates a significant contribution for the prediction purpose [4,9]. The Qh^2 ^values calculated for each dimension of the PLS and the sPLS showed that 2 dimensions were enough to capture the whole information for both PLS or sPLS. An equivalent coding for Y is the 2 column matrix of genotype and treatment factors that will be considered in the following.

The number of dimensions being fixed to 2, the optimal number of genes selected on each dimension (equal number of genes on both dimensions for the sake of simplicity), was determined with the RMSEP for both sPLS and PLS. The optimal result, *i.e. *the lowest RMSEP obtained was 10 genes on each dimension. In this case, the sPLS gave better predictions than PLS (not shown).

**Figure 3:**
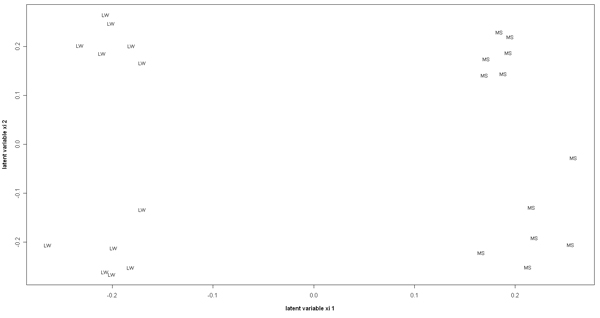
**Graphical representation of individuals with the two latent variables associated to the X data set**. The first axis (first latent variable) separates the two genotypes, while the second opposes the treatments.

Figure 3 displays the representation of the 24 individuals for the 2 dimensions of the 10+10 sPLS analysis and clearly shows a perfect separation between the 4 classes. The first axis separates the 2 genotypes and the second axis the treatment.

**Figure 4:**
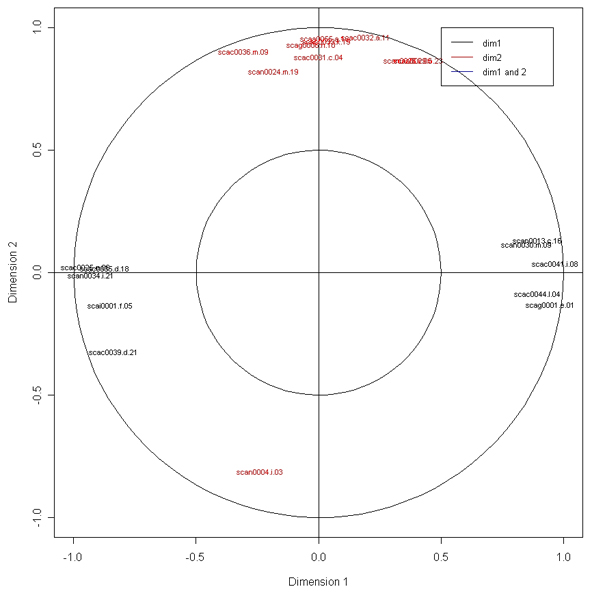
**Graphical representation of genes selected with sPLS and their correlation**. Genes clustered together indicate a high correlation between them. This figure can be combined with the interpretation of Figure 3: the genes in dark colour are predictive for the genotype effect (first axis) and the genes in red are linked with the treatment (second axis).

Figure 4 allows one to understand better the correlations between the selected genes in relation with the 2 latent components. In this graph, the coordinates of each selected gene are obtained by computing the correlation between the latent variables vectors and the whole data set X. These genes are then projected onto correlation circles where highly correlated genes cluster together and are closed to the larger circle. Interestingly, we observed that the 10 genes selected in dimension 1 were not the same as the 10 genes selected in dimension 2 (no overlap between the two gene lists). This may infer that each gene lists is related to a different effect in the data. Indeed, the interpretation of the axes deduced from Figure 3 can be combined to Figure 4 and we found that the genes on the right hand side were all significantly up regulated in Meishan breed (class MS). A closer look at the other sets of genes showed a good coherence between the differential analysis (t test) and the sPLS.

Figure 4 clearly illustrates the superiority of sPLS on PLS in terms of interpretability, as the PLS does not allow for variable selection.

The list of significantly expressed genes (t test) did not exactly match with the list of sPLS predictive genes. This shows that the information captured by the 2 approaches may bring complementary as well as relevant results.

## Discussion

Due to the clear structuring of the data, it is difficult to compare the performances of the statistical prediction approaches. A thorough biological interpretation of the results is now needed to validate the use of these methods.

In the case where *q *> 1 in the Y matrix, few other approaches have been developed for variable selection and integration of two-block data sets based on elastic net procedure [13] or shrinkage methods [14]. However, most of them focus on a canonical analysis, *i.e. *a symmetric relationship between the data sets, which is not the case in this study. The reader can refer to [15] (Canonical Correlation Analysis with Elastic Net) or [16] (Co-inertia analysis from [17]) for biological data sets.

In the case where *q *= 1, as performed with RF when we combined the phenotypes in one class vector, we find ourselves in a typical multiclass problem. Several approaches have been developed for feature selection, among them the reader can refer to Recursive Feature Elimination [18], Nearest-Shrunken Centroid [19] or Optimal Feature Weighting [20], that can deal with more than 2 classes.

## Conclusion

The differential analysis in [1], and the 2 predictive approaches presented here gave coherent, similar but complementary insights. On this data set however, expression patterns were so different in the 4 classes that the conclusions of the comparisons between the above statistical tools are not to be generalised.

In microarray data, the statistical criteria are often limited by the small number of samples. Therefore, it is strongly recommended to combine statistical assessments with a sound biological interpretation of the data, as was shown for example in [21]. They showed the importance of the interpretation of the results and found interesting complementarities between predictive approaches in several data sets, in terms of biological processes. Therefore, we also recommend the use of various predictive statistical tools when searching for biomarkers.

## Competing interests

The authors declare that they have no competing interests.

## Authors' contributions

KALC developed the sparse PLS method, and wrote the corresponding R code. CRG and MSC analysed the data set. All authors participated to the redaction work, read and approved the final manuscript.
